# Modifying lignin composition and xylan *O*-acetylation induces changes in cell wall composition, extractability, and digestibility

**DOI:** 10.1186/s13068-024-02513-5

**Published:** 2024-05-31

**Authors:** Aniket Anant Chaudhari, Anant Mohan Sharma, Lavi Rastogi, Bhagwat Prasad Dewangan, Raunak Sharma, Deepika Singh, Rajan Kumar Sah, Shouvik Das, Saikat Bhattacharjee, Ewa J. Mellerowicz, Prashant Anupama-Mohan Pawar

**Affiliations:** 1 Regional Centre for Biotechnology, Laboratory of Plant Cell Wall Biology, NCR Biotech Science, Cluster 3rd Milestone, Faridabad-Gurgaon Expressway, Faridabad-121001 Haryana, India; 2 Department of Forest Genetics and Plant Physiology, Umea Plant Science Centre, Swedish University of Agricultural Sciences, Umea, Sweden

**Keywords:** Acetyl Xylan Esterase (AXE), G Lignin, Saccharification, Xylose release

## Abstract

**Background:**

Lignin and xylan are important determinants of cell wall structure and lignocellulosic biomass digestibility. Genetic manipulations that individually modify either lignin or xylan structure improve polysaccharide digestibility. However, the effects of their simultaneous modifications have not been explored in a similar context. Here, both individual and combinatorial modification in xylan and lignin was studied by analysing the effect on plant cell wall properties, biotic stress responses and integrity sensing.

**Results:**

Arabidopsis plant co-harbouring mutation in FERULATE 5-HYDROXYLASE (*F5H*) and overexpressing *Aspergillus niger* acetyl xylan esterase (35S:*An*AXE1) were generated and displayed normal growth attributes with intact xylem architecture. This *fah1-2*/35S:*An*AXE1 cross was named as **h**ype**r G** lignin and **hyp**o**ac**etylated (HrGHypAc) line. The HrGHypAc plants showed increased crystalline cellulose content with enhanced digestibility after chemical and enzymatic pre-treatment. Moreover, both parents and HrGHypAc without and after pre-treating with glucuronyl esterase and alpha glucuronidase exhibited an increase in xylose release after xylanase digestion as compared to wild type. The de-pectinated fraction in HrGHypAc displayed elevated levels of xylan and cellulose. Furthermore, the transcriptomic analysis revealed differential expression in cell wall biosynthetic, transcription factors and wall-associated kinases genes implying the role of lignin and xylan modification on cellular regulatory processes.

**Conclusions:**

Simultaneous modification in xylan and lignin enhances cellulose content with improved saccharification efficiency. These modifications loosen cell wall complexity and hence resulted in enhanced xylose and xylobiose release with or without pretreatment after xylanase digestion in both parent and HrGHypAc. This study also revealed that the disruption of xylan and lignin structure is possible without compromising either growth and development or defense responses against *Pseudomonas syringae* infection.

**Supplementary Information:**

The online version contains supplementary material available at 10.1186/s13068-024-02513-5.

## Introduction

Biofuel production from lignocellulosic biomass can be a potential approach to mitigate effects of greenhouse gas emission because of excessive use of fossil fuels, and to produce a high-value biobased chemicals [10]. Second-generation bioethanol production along with platform chemicals could be an economically viable approach to sustain the biofuel industry if biomass recalcitrance is reduced using either a chemical or enzymatic approach. Both feedstock modifications, i.e., plant cell wall alteration during plant development and downstream processing, have been extensively researched to ease the process of biomass management [63]. Novel approaches are still necessary to understand lignocellulosic biomass and alter its structure by genetic engineering methods without affecting plant growth and yield.

A bulk of lignocellulosic biomass is mainly composed of plant secondary cell walls (SCWs) which are deposited inside the primary cell walls in xylem and sclerenchyma cells when their growth stops [32, 76]. Cellulose is a main component of SCW. It is a water-insoluble crystallite called microfibril which is composed of linear polymers made up of glucose subunits. Cellulose provides the structural integrity to plant cell wall. Xylan, the second most abundant SCW polysaccharide in angiosperms, is made up of xylose units often substituted with methylated or unmethylated glucuronic acid and acetyl groups. Lignin that makes up one-fourth of biomass is a phenolic polymer mainly present in the compound middle lamella (CML) and SCW layers in cells with secondary walls but typically absent in primary walls forming cells. Pectins, which are polysaccharides with a high galacturonic acid content, are mainly found in the primary cell walls of CML and help to maintain cell-to-cell adhesion [48]. All these above components interact either covalently or non-covalently to form a heterogeneous matrix which gives cell wall strength and rigidity [26, 67].

Xylan backbone is connected with β-1,4 linked xylose subunit [39]. Xylan in dicots is a branched polymer decorated with 2-*O*- and 3-*O*-acetyl and glucuronic acid or 4-*O*-methyl glucuronic acid which prevents self-aggregation [60]. In addition, the presence of an even substitution pattern favors the interaction between xylan and cellulose in dicots [24]. Although the direct interaction of lignin and acetyl groups is not known, it has been proposed that the presence of acetyl groups might limit the formation of ether type of lignin–carbohydrate complexes (LCCs) [22, 76]. Acetylation pattern also affects the pattern of glucuronosylation [13, 35] suggesting that the substitution of xylan is strictly regulated. Moreover, *O*-acetyl decorations prevent xylan degradation by glycosyl hydrolases during the saccharification process and contribute to the recalcitrance of the lignocellulosic biomass [73]. In addition, during the fermentation of lignocellulosic biomass, these *O*-acetyl groups are present in the form of acetic acid which might reduce fermentation efficiency. Therefore, altering acetyl content is an attractive target to improve the properties of the lignocellulosic biomass [57].

Previously, we have reported that Arabidopsis expressing fungal acetyl xylan esterase (AXE) from *Aspergillus niger* under the influence of constitutive as well as xylem-specific promoter showed an increase in esterase activity and reduced levels of xylan acetylation in both *Arabidopsis* and *Populus* [54, 55]. The transgenic lines showed increased xylan and cellulose digestibility with and without different pre-treatments. *Populus* AXE overexpressor lines had reduced S/G lignin composition and increased the lignin solubility in dioxane and water. Moreover, these lines exhibited elevated extraction of hot water-soluble lignin–carbohydrate fraction [56]. Similarly, overexpression of AXE from *Hypocrea jecorina* in *Populus* improved cellulose as well as xylan saccharification without a major effect on plant growth parameters [72]. In another approach, the downregulation of Reduced Wall Acetylation (*RWA*) genes reduced the overall xylan acetylation and improved saccharification [56]. All these *Populus* transgenic lines were grown in the field, and they maintained reduced acetylation after 5 years in the field with higher saccharification efficiency than wild-type trees [16, 59]. All these reports suggest that *in planta* reduction of xylan acetylation could be a viable strategy to improve lignocellulosic biomass properties.

Lignin monomers, i.e., monolignols, are synthesized in the cytoplasm through the phenylpropanoid pathway, transported and polymerized in the apoplast. Predominant monolignols, namely, coumaroyl alcohol (H), guaiacyl alcohol (G) and syringyl alcohol (S), participate in the formation of lignin [4]. Other non-conventional monomers can also be incorporated into lignin polymer making it a highly dynamic structure. Although lignin is well-known to hinder the bioprocessing of lignocellulosic biomass, lignin itself is valuable and can be utilized for making value-added chemicals or materials [28]. Many strategies of lignin modification have been tested before to improve the digestion of lignocellulosic biomass. Reducing lignin content by altering the expression of phenylpropanoid pathway genes in many cases improved digestibility, but it can negatively affect plant growth and total biomass yield [71]. However, Arabidopsis lignin composition mutants having either S, G or H type of lignin did not show any major growth defects [7, 44, 65]. Previous studies have shown that the glucose release during hot water pre-treatment is increased in both S- and G-lignin hyperaccumulating plants compared to wild type and mutant [44]. Arabidopsis with hyperaccumulation of H lignin showed increased saccharification efficiency with or without pre-treatment [37]. The glucose yield was found two times higher in H lignin plants as compared to wild-type plants [7].

Xylan and lignin are cross-linked in the cell wall matrix by covalent and van der Waals interactions. These interaction can also depend on lignin composition and S lignin can have more interaction points with xylan as compared to G lignin [29]. In addition, this can further influence xylan digestibility and extractability. These interactions might occur in the cell wall by coupling of xylan to the newly generated monolignol radicles with the formation of phenyl glycoside linkages [68]. It could also happen before the secretion of xylan to cell wall, as recently suggested for the γ-ester linkages [17]. The efficient conversion of lignocellulosic biomass into biofuel will be eased if the cross-linking between xylan and lignin is reduced [46, 47]. It might be through the catalytic or enzymatic breakdown of polysaccharide backbone, linkages between xylan and lignin or by altering the composition of responsible components [68]. Gene stacking could be a better approach to improve cell wall properties and understand the effect of polysaccharide digestibility and wall integrity. Altering the fibre-specific cell wall by gene stacking approach using the secondary cell wall-specific transcription factors to manipulate lignin biosynthesis has proved to be a good approach for improving lignocellulosic biomass [19]. Combinatorial post-synthetic modification by expressing fungal pectin and xylan acetyl esterase can alter plant immunity and cell wall integrity responses [66]. However, the effect on the plant cell wall and saccharification properties has not been studied using a gene stacking approach to alter both lignin and xylan.

In this study, we investigated the effect of reducing xylan acetylation and increasing G-lignin content in Arabidopsis, by analyzing cell wall composition and saccharification efficiency in the parents and the homozygotic hybrid between a line expressing acetyl xylan esterase from *Aspergillus niger* (*35S*:*An*AXE1) and *fah1-2* mutant enriched in G-lignin []. The newly generated homozygotic line called HyperGHypoAcetylated (HrGHypAc) exhibited an increase in cell wall carbohydrate content. The digestibility and extractability of the parents as well as HrGHypAc xylan wasincreased without any adverse effects on growth or after *Pseudomonas* infection.

## Results

### High G content and xylan hypo acetylation do not affect plant growth or biotic stress responses

**Fig. 1:**
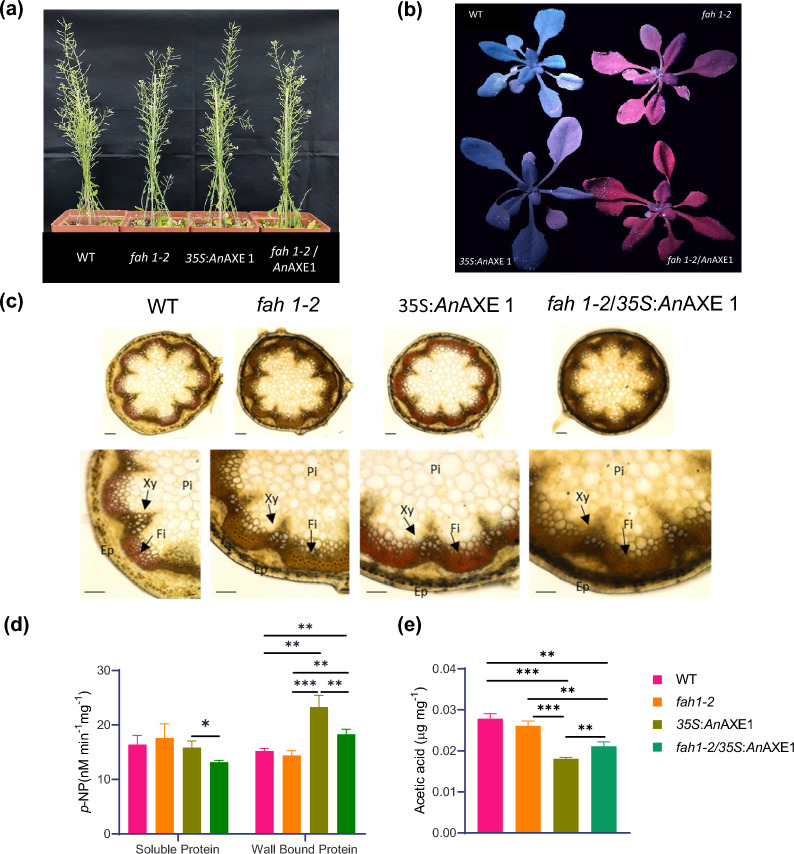
Morphological, anatomical and biochemical characterization.** a** Picture of 7-week-old plants, **b** Arabidopsis 4-week rosette leaf photographed under UV light. **c** Mäule staining of inflorescence stem transverse section. (Xy—xylem, Fi—interfascicular fibers, Pi—pith, Ep—epidermis and cortex) Scale Bar = 200 µm. **d** Esterase activity on soluble and wall-bound proteins extracted from 4-week-old leaf using *p*- nitrophenyl acetate as a substrate and released product 4-nitrophenol was analyzed by spectrophotometer. **e** Measurement of acetyl content on stem alcohol insoluble residue by Megazyme kit (K-ACET) after saponification treatment. Data represents mean ± SE,* n* = 3 biological replicates, Student’s *t* test at ***p ≤ 0.01, **p ≤ 0.05, * *p* ≤ 0.1 applied between genotypes

The *fah1-2* point mutation in FERULATE 5-HYDRXYLASE (*F5H*) gene leads to over-accumulation of guaicyl (G) lignin [11, 57], whereas the overexpression of acetyl xylan esterase from *Aspergillus niger* (*35S:An*AXE1) depletes xylan acetylation level in Arabidopsis [55]. To understand the effect of simultaneous G-lignin enrichment and acetyl-hypo-accumulation on plant cell wall properties and saccharification, we crossed the *fah1-2* mutant and the single-insert containing line D with *35S*:*An*AXE1 construct. The parent plants and their homozygous progeny were indistinguishable from the wild-type plants (Fig. 1a). To further confirm the presence of *fah1-2* mutation in the *F5H* gene in the obtained hybrid plants, we amplified the *F5H* fragment with gene-specific PCWL-58 and PCWL-59 primers (Additional file 2: Table S1). The amplicon obtained by amplifying *F5H* gene was digested with *MseI* restriction enzyme and the banding pattern after restriction showed the expected difference between wild type and *An*AXE1 showing 400 bp and 300 bp bands and *fah1-2* and *fah1-2*/*35S*:*An*AXE1 plants showing 300 bp, 275 bp and 125 bp bands (Additional file 1: Fig. S1a, b). Another typical chemotype of *fah1*-*2* mutant is the absence of sinapoyl malate []. Using a high-performance liquid chromatography coupled with diode array detector (HPLC–DAD) analysis of soluble metabolites from leaves revealed undetectable levels of sinapoyl malate in *fah1-2* and *fah1-2*/*35S*: *An*AXE1, whereas the wild-type and 35S:*An*AXE1 line D plants had detectable levels of this compound (Additional file 1: Fig. S1c). To further confirm the expected change in lignin composition in the hybrid, we exposed 4-week-old rosette leaves to ultraviolet (UV) light and we found that *fah1-2* and *fah1-2*/*35S*:*An*AXE1 plants showed red colour indicative of lack of sinapoyl malate level, whereas the wild-type and 35S:*An*AXE1 line D plants showed blue color (Fig. 1b). To demonstrate the lack of S-lignin in hybrid plants, we stained transverse sections of their inflorescence stem with Mȧule stain. We found that the interfascicular fibres stained red (positive for S-lignin) in the wild-type and 35S:*An*AXE1 plants and brown (absence of S-lignin) in *fah1-2* as shown by [21, 65] and *fah1-2*/*35S*:*An*AXE1 [58] (Fig. 1c).

Similarly, the presence of *An*AXE1 in the hybrid was confirmed by amplifying expected size band using *An*AXE1-specific primers PCWL-33 and PCWL-34 (Additional file 2: Table S1), whereas these amplicons were absent in for *fah1-2* and *wild type* (Additional file 1: Fig. S1d). The further analysis was performed on F2 and F3 generation plants which were positive for *An*AXE1 amplicon and also confirmed its expression by qPCR (Additional file 1: Fig. S1e, f). *An*AXE1 overexpressed lines showed an increase in esterase activity [55]. To evaluate this, we performed esterase activity assays on the leaves of the parent lines and the selected homozygotic hybrid line using *p*-nitrophenyl acetate as a substrate. Esterase activity in the wall-bound protein fraction was increased in the parent *35S*:*An*AXE1 line and in *fah1-2*/*35S*:*An*AXE1 homozygotic plants compared to the wild type and *fah1-2* mutants. (Fig. 1d). To further confirm a reduction in polysaccharide acetylation in the hybrid line because of the increase in esterase activity, we analysed cell wall acetyl content in alcohol-extracted wall residues (AIR) after saponification. As expected, we observed decreased acetyl content in *35S*:*An*AXE1 and *fah1-*2/*35S*:*An*AXE1 plants as compared to wild type and *fah1-2* mutant (Fig. 1e). These experiments confirmed expected genotypes and phenotypes in parents and *fah1-2*/*35S*:*An*AXE1. We hereafter referred to these crossed plants as **H**ype**rG Hyp**o **Ac**etylated line (HrGHypAc).

Morphological studies revealed that HrGHypAc plants were indistinguishable from wild-type plants. The xylem vessel cells were not collapsed **(**Fig. 1c**)** as in *irregular xyle*m (*irx)* cell wall mutants with compromised growth [69]. Often, changes in cell wall composition affect plant responses to biotic stress [45]. These could be because of weakening of wall, mechanical cues and change, cell wall-derived damage-associated molecular patterns (DAMPs) which can induce immunity. To evaluate innate immune responses of the HrGHypAc plants, we performed a quantitative pathogenesis assay. Individual parents were also tested similarly, with wild-type plants as controls. Fully expanded rosette leaves from the investigated plants were first infiltrated with *Pseudomonas syringae* DC 3000 *pv.* tomato strain (*PstDC3000*) suspension at a density of 5 × 10^4^ cfu/mL. The accumulation and growth of *PstDC3000* in the infiltrated leaves were evaluated at day 0 and day 3, post-infiltration. Simultaneously, we also determined the expression changes in defence-related marker genes, namely, *WRKY53*, *PR1*, *PAD3*, *LORE,* and *SERK i*n the infiltrated leaves at 0 and 3 dpi (days post-infection). Neither *fah1-2,* nor HrGHypAc plants showed any differences in bacterial accumulation at 3 dpi in comparison to the wild-type plants (Additional file 1: Fig. S1g). Interestingly, *fah1-2* plants showed higher upregulation of *WRKY53, PR1* and *PAD3* genes at 3 dpi compared to the wild type. Contrastingly, WRKY53 was down-regulated in *35S*:*An*AXE1 at day 3 dpi. In the HrGHypAc plants, the tested immune markers were wild type comparable indicating that genetically *FAH1* and over-expressed *AnAXE1* may function antagonistically on WRKY53 expression (Additional file 1: Fig. S1h). Since overall pathogenesis outcomes were unaffected in the tested plants for *Pseudomonas syringae* DC 3000 *pv.* tomato (*pstDC3000*) infection, it is implied that the contribution of these genes towards defence is not significant. Taken together, these data suggest that the HrGHypAc or the parental plants are not compromised in growth or defence.

### The total sugar content and Updegraff cellulose content is higher in HrGHypAc plants

**Fig. 2:**
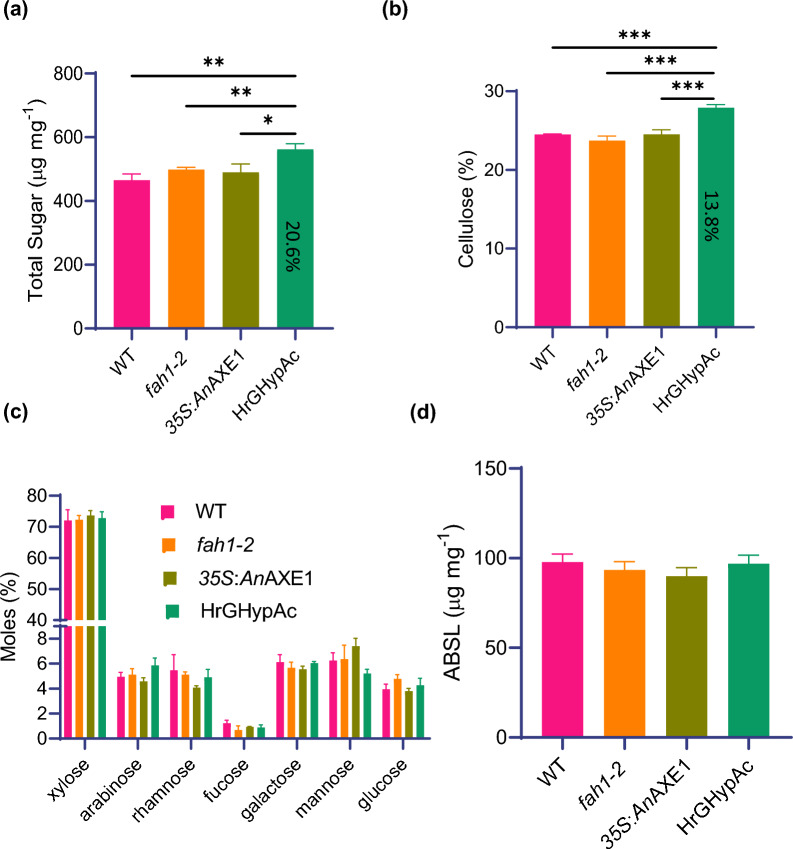
Plant cell wall composition on AIR samples prepared from Arabidopsis inflorescence stem.** a** Total sugar content estimated by phenol–sulphuric acid method. **b** Cellulose content by Updegraff’s method. **c** Non-cellulosic sugar composition through derivatization followed by gas chromatography**–**mass spectrometry. **d** Lignin content by acetyl bromide method, respectively. Data represent mean ± SE,* n* = 3 biological replicates, Student’s *t* test at ***p ≤ 0.01, **p ≤ 0.05, * *p* ≤ 0.1 applied between genotypes

To study the effect of simultaneous modification of lignin and xylan structure, we performed detailed cell wall characterization of alcohol insoluble residue (AIR) isolated from 7-week-old inflorescence stems of the above plants. Using the phenol sulphuric method, we analysed the total cell wall polysaccharide composition which was higher (+ 20%) in HrGHypAC as compared to wild-type or parent plants. (Fig. 2a). Furthermore, the content of crystalline cellulose by the Updegraff method was increased by 13% in HrGHypAc line as compared to either wild type or parent plants (Fig. 2b). Non-crystalline glucose originated from amorphous cellulose and hemicellulosic polysaccharide and other hemicellulosic sugars such as xylose, arabinose, rhamnose, fucose, galactose and mannose were similar in analysed plants (Fig. 2c). These findings suggests that the increase in total sugar content could be because of higher cellulose content. Another important component in the stem cell wall is lignin which was unchanged between investigated plants (Fig. 2d). Overall, these data suggest that HrGHypAc line contained increased total sugar, and crystalline cellulose content and with no major changes in lignin content or matrix sugar composition as compared to parent and wild type cell wall.

### HrGHypAc plants show improvement in xylan and cellulose digestibility

**Fig. 3:**
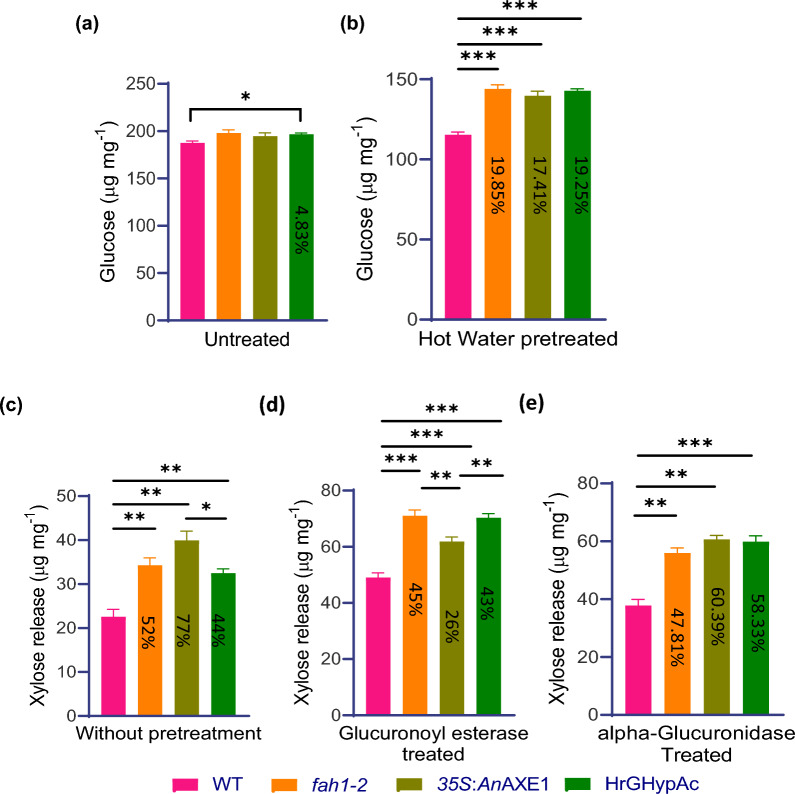
Saccharification on AIR of Arabidopsis inflorescence stem. **a** Saccharification using CTec2 commercial enzyme without any pre-treatment and **b** after hot water pre-treatment. The glucose content estimated through Megazyme GOD–POD assay megazyme kit (K-GLUC). Xylose release after **c** xylanase digestion **d** sequential glucuronoyl esterase and xylanase digestion **e** simultaneous alpha glucuronidase and xylanase digestion, respectively. Data represent mean ± SE,* n* = 3 replicates, Student’s *t* test at ***p ≤ 0.01, **p ≤ 0.05, * *p* ≤ 0.1 applied between genotypes. % difference representation is as compared to wild type plants

To understand the effect of modification in lignin monomer and xylan acetylation on biomass recalcitrance, we performed a polysaccharide digestibility assay with and without pre-treatment using AIR from the stems of parent genotypes, progeny and wild type. After digesting the untreated AIR of wild type, *fah1-2*, *35S*:*An*AXE1 and HrGHypAc with commercial enzyme blend containing cellulases and hemicellulases and by measuring the glucose release by GOD–POD assay [1], we detected moderately higher glucose (+ 4.38%) in the HrGHypAc compared to wild type, whereas the parent was comparable to wild type probably because of increase in total cellulose content (Fig. 3a). However, after hot water pre-treatment that can increase accessibility of polysaccharide degrading enzymes, the saccharification efficiency was increased in both the parents and HrGHypAc by 19.85%, 17.41%, and 19.25%, respectively, as compared to the wild type **(**Fig. 3b**)**. The glucose release was comparable in HrGHypAc and parent plants. Deacetylated xylan is more accessible to xylanases [55]. Therefore, AIR was digested with glycosyl hydrolase family 11 (GH11) xylanase without treatment, a higher level of xylose release was observed in parent and HrGHypAc plants as compared to the wild type. 3*5SAnAXE1* line exhibited a higher amount of xylose release as compared to the *fah1-2* and HrGHypAc plants (Fig. 3c).

Since glucuronoyl esterase (GE) breaks the γ-ester linkage between xylan and lignin [27, 49], AIR was treated with GE followed by endo-xylanases and xylose released by these treatments was increased by 45%, 26% and 43% in *fah1-2*, *35S*:*An*AXE1 and HrGHypAc, respectively, compared to wild type **(**Fig. 3d**)**. In addition, similar amount of xylose release was detected in *fah1-2* and HrGHypAc line but was higher than *35S*:*An*AXE1 parent. This effect could be because of more efficient xylan accessibility to xylanases for high G lignin plant as compared to low acetylated lines.

We also simultaneously digested AIR with a GH11 endo-xylanases and GH67 alpha glucuronidase (which hydrolyses glucuronic acid side-group on xylan chain) and found the xylose release increased by 47.81%, 60.39% and 58.33% in *fah1-2*, *35S*:*An*AXE1 and HrGHypAc, respectively, compared to wild type (Fig. 3e). Deacetylation of xylan often leads to changes in xylooligosaccharide (XOS) profiling after xylanase digestion [55]. Parent line *35S:AnAXE1* showed an increase in accumulation of non-acetylated xylobiose after xylanase digestion. To check effect on XOS pattern in *fah1-2* and HrGHypAc plants, xylanase digested AIR was analysed by MALDI–TOF. More abundant non-acetylated (Xyl_2_) and di-acetylated (Xyl_2_Ac_2__1_) were observed *35S:AnAXE1*, *fah1-2*, HrGHypAc as compared to wild type (Additional file 1**: Fig. S2).** Among these *35S:AnAXE1* had higher release as compared to *fah1-2* and *HrGHypAc*. Whereas longer chain XOS with tetra-XOS and penta-XOS with multiple acetyl group (Xyl4AC4, Xyl4Ac5, Xyl5Ac5) were less abundant in parents and HrGHypAc. This MALDI data correlated with an increase in accessibility for xylanases not only in lines with reduced acetylation but also in G lignin-rich lines when compared with previous results (Fig. 3c–e).

In summary, whereas only the HrGHypAc line showed an increase in glucose yield in saccharification without pre-treatment because of overall increase in cellulose content, all three lines, both parents and the HrGHypAc line, showed improved xylan digestibility as compared to wild type by endoxylanase after pre-treatment with hot water and either GE or alpha glucuronidase. In addition, altered XOS pattern in MALDI suggested easier accessibility of xylan to high-G lignin and hypoacetylated lines.

### Altered extractability and digestibility of xylan, cellulose and lignin in cell wall-modified lines

**Fig. 4:**
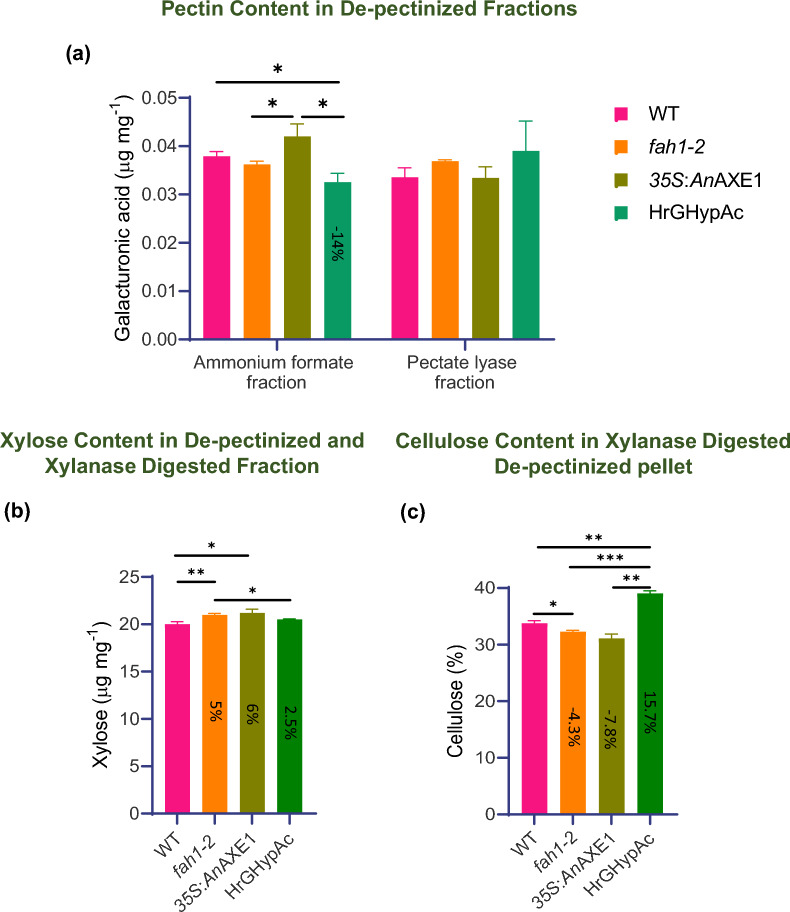
Sequential extraction and saccharification of AIR.** a** Galacturonic acid concentration estimated in by uronic acid assay kit (K-URONIC) from Megazyme in two pectin extracted fractions, namely, ammonium formate fraction and pectate lyase fraction. **b** Xylose content estimated in supernatant of de-pectinized pellet after GH11 xylanase digestion. **c.** Cellulose content determined in de-pectinized and xylanase digested pellet as described. Data represents mean ± SE,* n* = 3 technical replicates, Student’s *t* test at ***p ≤ 0.01, **p ≤ 0.05, * *p* ≤ 0.1 applied between genotypes. % difference representation is as compared to wild-type plants

Lignin and xylan modification can alter its interaction with pectin and consequently can influence polysaccharide digestibility [8, 41, 43]. To study further the effect of the genotypes (*fah1-2*, *35S*:*An*AXE1 and HrGHypAc) on the accessibility of xylan to a GH11 endoxylanase, the AIR was treated with ammonium formate to solubilise pectin and the remaining pellet was digested with pectate lyase to completely remove the residual homogalacturonan. The galacturonic acid was analysed in both pectin-rich ammonium formate extract and in pectate lyase fraction. HrGHypAc plants showed a decrease in galacturonic acid level by 14% as compared to the wild type in the ammonium formate extract but it was similar in wild type, parent lines and their progeny in the pectate lyase fraction (Fig. 4a). The de-pectinated pellet obtained in above assay was digested with a GH11 endoxylanase and the total xylose released by the enzyme was measured in the supernatant. Significantly higher xylose content than wild type (5%, 6% and 2.5% in *fah1-2*, *35S*:*An*AXE1 and the HrGHypAc, respectively) were detected in this assay (Fig. 4b). Subsequently, we measured cellulose content in de-pectinized xylanase digested samples, found it significantly higher by 49%, 41% and 55.5% in *fah1-2*, *35S*:*An*AXE1 and the HrGHypAc, respectively, as compared to wild type (Fig. 4c). To understand the effect of the genotypes on lignin content in de-pectinized samples and in de-pectinized xylanase-digested samples, we measured acetyl bromide soluble lignin (ABSL) in these samples. In de-pectinized samples, ABSL was significantly increased in the HrGHypAc line by 7% as compared to wild-type and parent lines (Additional file 1: Fig. S3a). In the de-pectinized xylanase-treated samples, *fah1-2* and *35S*:*An*AXE1 lines showed 13% and 9.5% decreased lignin content, respectively, and the HrGHypAc line showed similar lignin content as wild-type plants (Additional file 1: Fig. S3b). We also measured ABSL content after saccharification and found that it was decreased by 5.7%, 10.4% and 15%, respectively, in *fah1-2, 35S*:*An*AXE1 and HrGHypAc lines (Additional file 1: Fig. S3c). Lower lignin content after each fraction extraction probably suggests more lignin attached to pectin is removed in the first ammonium formate fraction. To further understand the effect of genotypes on xylan extractability, we sequentially extracted cell walls with ammonium formate and KOH and analysed the xylan content in 1 M and 4 M KOH fractions using LM10 antibody. The xylan signal was significantly increased in *fah1-2*, *An*AXE1, and the HrGHypAc plants, respectively, as compared to wild type in combined 1 M and 4 M KOH fractions (Additional file 1: Fig. S3d). The extractability was higher in *35S*:*An*AXE1 among all tested genotypes. All these cell wall digestibility and extractability experiments revealed that both HrGHypAc and parental plants show altered xylan digestibility and lignin extractability after sequential extraction.

### HrGHypAc plants show increased expression of CESA, THESUS1 and WAK genes

**Fig. 5:**
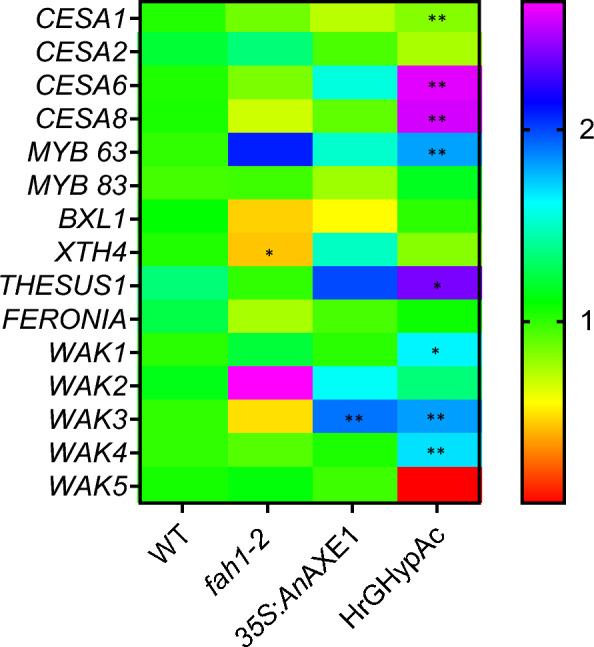
Heatmap representing qPCR-based expression analysis of genes involved in cell wall synthesis defense and integrity. Quantification of the cellulose biosynthesis genes (*CESA1, CESA2, CESA6, CESA8*), transcription factor related to the secondary cell wall biosynthesis (*MYB63* and *MYB83)*, xylan modulating genes, receptor-like kinases (*BXL1, XTH4*, *WAK1–WAK5, THESUE1, FERONIA*) by qRT-PCR. Data represents mean,* n* = 3 biological replicates, Student’s *t* test at ***p ≤ 0.01, **p ≤ 0.05, * *p* ≤ 0.1

To correlate the changes in plant cell wall properties in parental and HrGHypAc plants, we evaluated the expression of the key cell wall-associated genes. Cellulose Synthases (*CESAs*) in the rosette complexes present in the plasma membrane are responsible for the cellulose polymer biosynthesis [36]. When tested for relative expression levels of various CESAs transcripts, primary - and secondary cell wall-specific CESA6 and CESA8 (+ 2.6 and + 2.5-fold) mRNAs were significantly upregulated, respectively whereas CESA1 was lower (-19.64%) in HrGHypAc plants than wild type **(**Fig. 5**)**. No significant change in fold change expression was found for CESA2 among tested plants. The expression of secondary cell wall biosynthesis transcriptional activator *MYB83* [31] and the lignin biosynthesis pathway transcriptional activator *MYB63* [77] were also higher in the HrGHypAc line when compared to wild type **(**Fig. 5**).** Expression levels of xylan modifying enzyme β-xylosidase 1 (BXL1) [23] and xyloglucan-modifying enzyme *XTH4* (Xyloglucan endotransglucosylase/hydrolase) [33] were unchanged except *XTH4* in *fah1-2* compared to the wild type (Fig. 5). *THESUS1* (THE1) encoding receptor-like kinase involved in sensing defective cell wall growth and development, and a related gene *FERONIA* (*FER*) implicated maintenance of in pollen tube cell wall integrity [11] were upregulated in the HrGHypAc by 78.61%, or unchanged compared to wild type (Fig. 5). Similar to THE1 and FER, Wall-associated kinases (WAKs) function as cell wall integrity sensing receptors associated with pectin and have an affinity for pectin oligomers which may be released during cell wall degradation [2]. HrGHypAc showed upregulation of several *WAKs* (*WAK1, 3 and 4*) (Fig. 5). This transcriptomic data suggested secondary cell wall-specific genes and receptor-like kinases are differentially regulated in mainly in HrGHypAc plants.

## Discussion

According to a recent report, the affinity of lignin to xylan is more as compared to lignin to cellulose [29]. Lignin–cellulose interactions are bridged by xylan structure, suggesting the importance of lignin–xylan interaction in determining secondary cell wall structure and lignocellulosic biomass properties. Therefore, in this study, we analysed cell wall digestibility and extractability in hypoacetylated lines and high G lines and in combination which was not done before. In our previous study, we reported that overexpression of AXE from carbohydrate esterase (CE) family 1 improves saccharification efficiency and ethanol production in Arabidopsis [55]. However, the xylan digestibility and extractability were not extensively studied in either of the parents. Therefore, we crossed *An*AXE1 expressed line D with G lignin hyperabundant *fah1-2* mutant. The homozygous hybrid HrGHypAc plants were growing normally in controlled growth chamber conditions, because the xylem vessel structure was intact. This also suggests that lignin and xylan interactions in HrGHypAc plants give stable cell wall structure [9]. In addition, HrGHypAc cell wall showed an increase in total polysaccharide sugar content because of an increase in Updegraff cellulose content. C*ESAs* are responsible for cellulose synthesis and the expression of two of these genes were significantly upregulated in HrGHypAc plants (Fig. 5). This increase in cellulose level and other positive effect on cell wall digestibility and extractability was observed in both parents and HrGHypAc. In addition, the overall growth was comparable suggesting, such alterations in important bioenergy trees or crops can be viable approach to alter lignocellulosic biomass structure [55]. We also tested the effect of the pathogen *Pseudomonas syringae* DC3000 *pv.* tomato to understand whether plant defense was compromised and it was not affected either in parents or HrGHypAc plants (Additional file 1: Fig. S1g, h). In addition, the expression of defence-related marker genes in HrGHypAc plants were comparable between the wild-type and HrGHypAc plants. We have tested this only in *Psedomonas* but this study can be further extended to understand interaction with other bacterial and fungal pathogens. The glucose release was increased by 4.83% without pre-treatment only in HrGHypAc, this increase may be observed because of the increased cellulose content in HrGHypAc (Fig. 3a). Similarly, after hot water treatment, the glucose release was hiked by 19.25% in HrGHypAc plant. *35S*:*An*AXE1 overexpression showed 17.14% increase in saccharification efficiency after hot water treatment. In addition, the *fah1-2* showed 19.85% increase in saccharification (Fig. 3b). Importantly, HrGHypAc plants showed an increase in saccharification even after hot water treatment similar level to *fah1-2* line. Similar results were observed by [54, 56] in Arabidopsis stem expressing fungal acetyl xylan esterase after hot water pre-treatment. In all parents and HrGHypAc plants, hot water treatment might have exposed more cellulose surface, facilitating access for cellulolytic enzymes. Parents and HrGHypAc plants xylan was digested more easily than wild-type plants xylan without any pre-treatment **(**Fig. 3c**)**. *35S:AnAXE1* showed maximum xylose release as compared to *fah1-2* and *HrGHypAc* because of stronger deacetylation in *35S:AnAXE1* (Fig. 1e). We also found a significant effect of genotypes on xylanase digestion when digested with α-glucuronidase and glucuronyl esterase (Fig. 3d, e). We used these two enzymes, because they are responsible for breaking the putative connection between xylan and lignin [34, 61]. In wild type scenario, the addition of α-glucuronidase and glucuronyl esterase has increased xylose release in combination with xylanases than alone with xylanases (Fig. 3c, d). This emphasized further that lignin carbohydrate complexes influence biomass recalcitrance. In addition, reducing the complexity of xylan and xylan–lignin through glucuronidase and esterase can be further explored by analysing undigested and digested monosugars or oligosaccharides. As the activity of these enzymes is often hindered because of individual polysaccharide properties and interaction between cell wall components. Therefore, more studies are necessary to understand the interaction between lignin and hydrolytic enzymes in plants with specific lignin composition and altered xylan structure. This will further emphasize the advantages of such modification and the addition of enzymes which break covalent linkages in cell walls [42] (Fig. 3d, e**).**

Improvement in xylanase digestibility with and without αtreatment can be explained in plants with lower xylan acetylation (*35S:AnAXE1* and HrGHypAc) but it is surprising for *fah1-2* plants as their lignocellulose is known to be resistant for cellulase digestion [37]. In addition, MALDI data revealed an increase in shortest XOS, i.e., xylobiose in *fah1-2* plants, suggesting its xylan is more accessible than wild type. The possible explanation could be lower interaction of xylan with G lignin as compared –S-lignin, because this interaction depends of the *O*-methyl group present on the aromatic phenyl ring [29]. However, this needs to be further investigated by studying G-lignin and xylanase interaction during the saccharification process with and without the pre-treatment in species with extreme lignin composition [29, 38]. Also, it was shown that lignin–cellulase interaction can influence the saccharification process [42]. Furthermore, we analyzed changes in cell wall extractability and found lower pectin galacturonic acid levels in ammonium formate fraction of HrGHypAc as compared to wild type and parents (Fig. 4a). Xylanase digested de-pectinated pellet showed an increase in xylose release both in parents and HrGHypAc (Fig. 4b). The cellulose content measured in pellets was elevated in *35S*:*An*AXE1, *fah1-2* and HrGHypAc lines. In an independent experiment, we found that xylan epitopes were more abundant in KOH fractions of parents and HrGHypAc lines than in wild type, indicating increased xylan extractability (Additional file 1**: **Fig. 3d). The xylan extractability was higher also in both parents as compared to wild type. Lignin was reduced in xylanase and cellulase digested pellet suggesting changes in plant cell wall structure (Additional file 1**: **Fig. 3b, c). All these results suggest that the pectin–xylan connection is loosened in parental and HrGHypAc plants. Similarly, *irregular xylem (irx) 9* and *irx15 irx15L* showed an increase in pectin and xylan extractability [14]. It is also well known that pectin modification improves polysaccharide digestibility and extractability [5, 6]. More cell wall analytical studies are needed to dissect the mechanism of improvement in selective digestibility and extractability in both lignin and xylan-modified lines. Overall, the lignocellulose of high G lignin and deacetylated plants responds positively to both chemical and enzymatic treatment which is beneficial trait in biofuel industry.

The changes in cell wall generally perceived by plasma membrane receptors, that further transduce the signal which reinforces or weaken the cell wall or impacts the immune response which is known as plant cell wall integrity (CWI) maintenance [51]. Cell wall elicitors was isolated from *fah1-2* induced the expression of defense-related genes mostly because of the abundance of pectin elicitors [40]. *An*AXE1 plants were resistant to *Hyaloperonospora arabidopsidis* infection and had altered defence responses [55]. This suggests alteration in CWI responses in parent plants. Although we did not see growth defect in the hypoacetylated G lignin-rich line, the cell wall integrity signalling might have been altered as changes in cell wall extractability and digestibility are clearly observed in these plants. In qPCR data, we found that *THESUS1* (THE1), *WALL ASSOCIATED KINASES 1 (WAK1)*, *WAK3* and *WAK4* expressions higher in the HrGHypAc line but were similar to wild type in parent plants **(**Fig. 5**)**. *THE1* belongs receptor-like kinase (RLK) family which was identified in suppressor screening of cellulose-deficient mutant [25]. WAK is a five-member family in Arabidopsis and *WAK1* binds to oligo-galacturonides [15]. Perturbation in lignin and xylan acetylation might activate cell wall integrity sensing by *THE*1 or *WAK1,* which could alter cell wall structure [3, 40, 74]. Complete transcriptomic and proteomics analysis and elicitor studies in parents and HrGHypAc might reveal differential regulation of plant cell integrity sensing upon xylan and lignin modification.

Based on these results, we propose a model of how abundant G lignin and hypoacetylated xylan can influence polysaccharide digestibility. A probable decrease in proximity between G-lignin and xylan improves xylan accessibility in *fah1-2* plants [29]. On the other hand, it is well known that chemical and enzymatic removal of the acetyl group in a controlled manner from xylan improves its saccharification, which is also the case for *35S:AnAXE1* plants. The HrGHypAc lines showed an increase in accessibility of xylan along with a slight increase in cellulose content because of both hypoacetylation and higher G lignin (Additional file 1: Fig. S4).

## Conclusions

Hyperaccumulation of the G lignin in combination with the post-synthetic reduction in xylan acetylation increases cellulose content. An increase in xylose and xylobiose release after xylanase treatment in hypoacetylated and high G lignin-rich plants was revealed without pre-treatment. Subtle changes in the cell wall of parents and HrGHypAc were evident from changes in extractability in cellulose, xylan and pectin. Changes in the transcription of cell wall-associated biosynthetic and signalling genes suggest activation of plant cell wall integrity sensing, which seems more prominent when both the lignin and xylan structures are modified. Overall, this study revealed that simultaneous or individual modification of xylan and lignin could be tested in other bioenergy crops to improve cell digestibility and extractability.

## Methods

### Plant growth condition and genotyping

Homozygous plants of *fah1-2* [] and *35S*:*An*AXE1-Line D [55] were grown in a plant walk-in growth chamber under 16-h-light/8-h-dark photoperiod at 22 °C. The mutation in *fah1*-*2* was genotyped by amplifying the F5H gene using primers PCWL-58 and PCWL-59 (Additional file2: Table S1) followed by digestion with *MseI* (R0525S, New England Biolabs, UK). *An*AXE1 gene was confirmed using primers PCWL-33 and PCWL-34 (Additional file2: Table S1). The* fah1*-*2* mutant was further confirmed by UV phenotyping, homozygous *fah1*-*2* mutant lacked sinapoyl malate and exhibit red colour under UV. Mature parent plants in flowering stage were then crossed using forceps and magnifier to get the F1 progenies. After harvesting the F1 seeds the progenies were screened for F5H and *An*AXE gene as described above. The screening was continued for self-pollinated plants up to 3 (F4) generations and then the plants were used for further analysis.

### Detection by sinapoyl malate by HPLC

Three-week-old Arabidopsis leaf was processed with 70% methanol, incubated at 65 °C for 1 h and the sample were run on a C18 column (Waters, SunFire C18, 2.5 µm, 3.0X75 mm column). The gradient was set from 98 to 80% of 0.1% formic acid for 20 min with acetonitrile and then reversed back to 98% up to 35 min on Agilent 1260 Infinity II HPLC–DAD system. The injection volume was 10 µl with a column temperature of 40 °C. The diode array detector was set at 330 nm with 1 nm bandwidth [30, 42].

#### Mäule staining

Six-week-old Arabidopsis inflorescence stem were stained by Mäule reagent as described by [58]. Briefly handmade stem sections were treated with 0.5% potassium permanganate for 5 min, washed with water and dipped in 3.5% hydrochloric acid (HCl) to remove excess colour. After removing HCl, the section was treated with ammonium hydroxide and immediately observed under a Nikon ECLIPSE T*i* fluorescence microscope with 10X and 20X magnification.

#### *Total RNA *extraction* and qRT-PCR*

Total RNA was extracted from fresh main stems by the Trizol method (Cat#15,596,018, Thermo Fisher Scientific—Invitrogen). DNA contamination was removed by DNAse treatment using the kit (AM1907, Thermo Fisher Scientific, Lithuania). cDNA was synthesized from 1 µg of DNAse treated RNA with PrimeScript TAKARA cDNA synthesis kit. (Takara, 6110A). qPCR was performed using HOT FIREPol EvaGreen qPCR Mix Plus (ROX) (Solis Biodyne, 08–24-00001, Estonia) master mix on QuantStudio- 6 Flex Real Time PCR machine for gene of interest and reference gene (Additional file2: Table S1). The expression was quantified using the ΔΔCt method.

#### *Esterase activity *assay

Esterase activity was determined by extracting soluble and wall-bound protein fractions in phosphate buffer [6]. The esterase activity was carried out using *p-*nitrophenyl acetate as a substrate (Sigma, N8130, Switzerland). The enzyme reaction mixture was incubated for 2 h at 37 °C and the release of *p*-nitrophenol (Sigma, 1048, United States) was quantified by spectrophotometry at 400 nm. The specific esterase activity was estimated by calculating nM per minute per mg of total protein using the *p*-nitrophenol standard curve.

#### *Pseudomonas *infection* study*

For *Pseudomonas syringae* DC 3000 *pv.* tomato (*pstDC3000*) plants were grown at short day condition, i.e., 8/16-h photoperiod with 70% humidity. Plants were grown for 1 month and leaves from fully expanded rosettes were used for infection. Leaves were infiltrated with bacterial suspension in 10 mM MgCl_2_ at concentration of 5 × 10^4^ CFU ml^−1^ using needle less syringe. Bacterial infection was measured on 0 and 3rd dpi by harvesting leaf discs of constant diameter and macerating them in 10 mM MgCl_2_ and inoculating their serially diluted suspension on *Pseudomonas* agar plates with antibiotic selections. Bacterial infection in each genotype were then estimated through number of colonies and expressed as Log_10_CFU cm^−2.^ [64].

#### *Preparation of stem *powder* for cell wall analysis*

The inflorescence stem of 10 cm was collected from a completely dried Arabidopsis plant and homogeneous fine powder was prepared using Qiagen TissueLyserII for further cell wall analysis. The stem powder was treated for 30 min at 70 °C in 80% ethanol prepared in a 4 mM HEPES buffer. After treatment, sample was washed with 70% ethanol, trichloromethane: methanol (1:1) and acetone sequentially. The final pellet was dried in a desiccator overnight and this alcohol insoluble residue (AIR) was used for plant cell wall composition analysis.

### Cell wall composition analysis

#### Quantification of acetyl content

Acetic acid content was estimated by treating 1 mg AIR with 1 M NaOH. Treated samples were then neutralized with equimolar HCl. Samples were diluted and the mixture was used for acetic acid quantification by Megazyme kit (Megazyme, K-ACET, Ireland).

#### Total sugar content

Total sugar was estimated by the phenol sulphuric acid method. AIR was suspended in Milli-Q water at a concentration of 0.5 mg/ml. The reaction was set up in a glass tube by adding 100 μl of 5% (v/v) phenol solution and 100 μl of sample suspension. 500 μl of concentrated sulphuric acid was added to the reaction, vortex thoroughly, and incubated for 20 min in fume hood. After the completion of incubation, the absorbance of reaction mixture was taken at 490 nm. The concentration of total sugar in the samples was estimated using glucose standard curve.

#### Cellulose content quantification

Cellulose estimation was performed on 1 mg AIR by performing nitric acid: acetic acid: water treatment. The treated samples were then acid hydrolysed to convert cellulose polymer into glucose monomer. The glucose content was then quantified by an Anthrone (96,476, SRL, India) assay[70].

#### Hemicellulosic mono sugar composition analysis by GC–MS

2 mg of AIR samples were treated with 2 M trifluoroacetic acid (208–02741, Wako, Japan) at 121 °C for 90 min, then centrifuged at 10,000 rpm for 10 min and supernatant was collected in glass tubes. TFA was evaporated from supernatant under a stream of nitrogen gas and treated with isopropanol, three times and evaporated. Then, the dried fractions were re-dissolved in methoxyamine hydrochloride (139–18,071, Wako, Japan) prepared in pyridine (163–05326, Wako, Japan) and incubated for 90 min at 37 °C. These fractions were then derivatized at 37 °C with N-methyl–N-trimethylsilyltrifluoroacetamide (MSTFA) (130–17881, Wako, Japan) for 30 min. 100 µl of derivatized samples were then run on GC–MS. Inositol was used as internal standard in each sample.

#### Lignin content quantification

1 mg of AIR was treated with 25% acetyl bromide (135,968, Sigma-Aldrich, USA) prepared in acetic acid at 50  °C for 2 h with intermittent shaking. 2 M NaOH and freshly prepared 0.5 M Hydroxylamine hydrochloride (159,417, Sigma-Aldrich, China) were then mixed to samples. Samples were diluted with acetic acid and absorbance was taken at 280 nm. [20].

#### Xylo-Oligosaccharides Mass Profiling (OLIMP) through MALDI–TOF–MS

AIR sample was incubated with xylanase (E-XYLP, Megazyme, Ireland) for 48 h at 60ͦC [12]. The supernatant was separated and passed through a porous graphite column (60,106–303, Thermo Scientific, USA) and eluted neutral xylooligosaccharide fraction in 50% acetonitrile under Visiprep™ SPE Vacuum Manifold, the fraction was freeze dried, dissolved in water and used for MALDI analysis. The fraction was placed on a MALDI plate and crystallized with dihydro benzoic acid under a stream of air. The crystallized sample was analysed using the 5800 MALDI–TOF/TOF analyzer (AB SCIEX) and MS analysis was done using 4000 Series Explorer software, version 4.0 (AB SCIEX). The Instrument was operated in positive ion mode, and external calibration was performed using a calibration mixture 1 from a mass calibration standard kit (AB SCIEX). The laser power was set between 3100 and 3500 for MS acquisition. MS spectra were acquired between 800 and 4000 m/z. The peaks between 249 and 1004 m/z were annotated according to [12] and relative percentage intensity for each peak was calculated.

#### Cell wall digestibility analysis

Saccharification of untreated, hot-water treated AIR was performed using cellulase enzyme blend (SAE0020-50ML, Sigma, China). The enzyme concentration and incubation period were first standardized for Arabidopsis stem biomass according to the dosage guidelines suggested by Novozymes (*FUEL ETHANOL APPLICATION SHEET Cellic® CTec2 and HTec2-Enzymes for Hydrolysis of Lignocellulosic Materials*, n.d.). 15% w/w of cellulose enzyme was used for each sample. Samples were incubated at 50 °C for 48 h in a dry bath with constant stirring. After incubation, the samples were centrifuged at 16,000 rpm speed for 5 min. The hydrolysed supernatant was analysed for the glucose content with the help of the GOD–POD kit. (K-GLUC, Megazyme, Ireland). AIR was pre-treated with hot water at 90 °C for 30 min. After that, the sample was centrifuged and the supernatant was collected and stored. Pellet was washed with Milli Q water, acetone and dried in vacuum desiccator. Saccharification on pre-treated samples was done as described above.

#### Xylanase digestibility prior and after/with glucuronoyl esterase and α-glucuronidase treatment

Xylanase digestion was performed by mixing AIR with xylanase GH11 (SRL, 13,814) prepared in reaction buffer, i.e., acetate buffer (pH 4.5) equivalent to approximate 13 U/ reaction. The samples were then incubated in acetate buffer (pH 4.5) at 50 °C for 6 h. After incubation the xylose content from supernatant was estimated using megazyme kit (K-XYLOSE, Megazyme, Ireland).

Xylanase digestibility of the AIR was checked after the removal of the glucuronic acid and the glucuronoyl ester linkages the AIR was partially treated with the glucuronoyl esterase and with the α-glucuronidase separately. The reaction for the glucuronoyl esterase (Megazyme, E-GERF, Ireland) treatment was set with the 0.1 M sodium phosphate buffer (pH 6.5) and 2 µl/mg AIR enzyme. The reaction was incubated at 40 °C for 6 h. For α-glucuronidase (Megazyme, E-AGUBS, Ireland) treatment, the AIR was incubated with 0.1 M MOPS buffer (pH 7) containing 2 µl/mg AIR α-glucuronidase enzyme and xylanase (GH11) synergistically at 40 °C for 6 h. The samples pellets were washed with MilliQ water two times, acetone and dried in the vacuum desiccator. Dried pellet after glucuronoyl esterase was digested with the GH11 xylanase as described before for 6 h. Xylose content was analysed from the supernatant of the digestion reactions using the xylose assay kit (K-XYLOSE, Megazyme, Ireland).

#### Sequential extraction of cell wall components and xylan quantification through immunodetection

For sequential extraction we followed protocol as per [62]. The stems AIR was first treated with 50 mM ammonium formate at 37 °C for 24 h, centrifuged and the supernatant collected. Then the remaining pellet was digested with desalted pectate lyase in 50 mM tris HCl buffer (pH 8) at 40 °C for 24 h. Again, the samples were centrifuged and supernatant was collected. Both the ammonium formate and pectate lyase supernatants were lyophilized and re-dissolved in water. The pellet was washed with water, acetone and dried as described before. Galacturonic acid from the above two fractions were estimated by kit (Megazyme, K-URONIC, Ireland). The washed pellet was then subjected to the GH11 xylanase digestion and xylose quantification was as described earlier. Pellet was washed with water and acetone and dried in a vacuum. The cellulose content of the xylanase-digested pellet was estimated by Updegraff's method. All the remaining pellets after pectin extraction, xylanase (GH11) digestion and saccharification were analysed for the ABSL content as described.

#### Glycan antibody profiling

Different fractions of cell wall components were isolated through sequential extraction using ammonium oxalate, sodium carbonate, 1 M KOH, and 4 M KOH. Total sugar content was estimated by the phenol–sulphuric acid method as described before, and all wall extract samples were diluted to a sugar concentration of 0.25 μg/mL. in ELISA plate and evaporated at 37 °C. Nonspecific sites in the coated ELISA plates were blocked by incubating with blocking buffer and room temperature. 50 μL xylan-specific primary antibodies LM10 (1:20 dilution) (AS184206 Agrisera, Sweden) were dispensed into each well and the plate was incubated for an hour at room temperature. The primary antibody was washed with the wash buffer and 50 μL of Anti-rat secondary antibodies (1:5000 dilution) (A9542, Sigma-Aldrich, Germany) was added to each well and incubated for an hour. Wells were washed with the washing buffer, 50 μL TMB substrate solution was added into each well and incubated for 20 min at 37 °C, reaction was stopped and monitored absorbance at 488 nm. The standard curve of xylan (Birchwood) (5 to 125 ng/ml) was prepared and used for xylan estimation in samples [52].

#### Calculations and Statistics

All the calculations and statistics were performed in Microsoft excel using the student’s *t* test.

### Supplementary Information

**Additional file 1: Figure S1. a** Gene-specific amplification of F5H gene. **b** MseI digestion of F5H gene amplicon, **c** Qualitative analysis through HPLC–DAD of sinapoyl malate in methanolic extract of fresh leaves**. d** Gene-specific amplification of AnAXE gene. **e** Genotyping of F2 and F3 generation representatives of HrGHypAc line positive for AnAXE. **f** Expression analysis of *An*AXE in *35S*:*An*AXE and HrGHypAc. **g** Defense response of parents and HrGHypAc to the infection of *Pst DC3000* with respect to the wild type in bacterial accumulation of the infiltrated leaves. **h** Expression of defense-related genes in day 0 and day 3 leaf of *Pst DC3000* infected samples. Data represents mean,* n* = 3 biological replicates, Student’s *t* test at ***p ≤ 0.01, **p ≤ 0.05, * *p* ≤ 0.1. **Figure S2. a** MALDI–TOF–MS. **a** Representation of relative intensity of xylo-oligosaccharide after xylanases digestion calculated from raw spectra of **b** wild type, **c** *fah1-2,* **d** *35S*:*An*AXE1,** and e** HrGHypAc, respectively. **Figure S3. a** ABSL content after pectin extraction in sequential extraction, **b** ABSL content after xylanase digestion in sequential extraction, **c** ABSL content after saccharification in sequential extraction. **d** Xylose extractability through glycome antibody profiling in 1 M KOH, 4 M KOH samples and their combined representation. Data represents mean ± SE,* n* = 3 biological replicates, Student’s *t* test at ***p ≤ 0.01, **p ≤ 0.05, * *p* ≤ 0.1. **Figure S4.** Proposed model based on xylan digestibility experiment that explains WT xylan accessibility is limited because of hyper-acetylation, and the presence of abundant S lignin. *fah1-2* and 35S:AnAXE1 xylan accessibility is increased because of less proximity between lignin–xylan and deacetylation of xylan, respectively. Enhancement in HrGHypAc xylan accessibility is because of the slight decrease in xylan acetylation and G lignin abundance. HrGHypAc lines deposits more cellulose because of change in cell wall integrity.

**Additional file 2: Table S1.** Primers used for genotyping of *fah1-2* and *An*AXE1 and gene expression analysis.

## Data Availability

All the data regarding this manuscript is provided in the form of figures and additional file.
