# Supplementary material for: Modifying lignin composition and xylan O-acetylation induces changes in cell wall composition, extractability, and digestibility
Source: Biotechnol Biofuels Bioprod. 2024 May 31;17:73. doi: 10.1186/s13068-024-02513-5 (PMC11141020; doi:10.1186/s13068-024-02513-5)
Supplement: Supplementary file 1 — Additional file 1: Figure S1. a Gene-specific amplification of F5H gene. b MseI digestion of F5H gene amplicon, c Qualitative analysis through HPLC–DAD of sinapoyl malate in methanolic extract of fresh leaves. d Gene-specific amplification of AnAXE gene. e Genotyping of F2 and F3 generation representatives of HrGHypAc line positive for AnAXE. f Expression analysis of AnAXE in 35S:AnAXE and HrGHypAc. g Defense response of parents and HrGHypAc to the infection of Pst DC3000 with respect to the wild type in bacterial accumulation of the infiltrated leaves. h Expression of defense-related genes in day 0 and day 3 leaf of Pst DC3000 infected samples. Data represents mean, n = 3 biological replicates, Student’s t test at ***p ≤ 0.01, **p ≤ 0.05, * p ≤ 0.1. Figure S2. a MALDI–TOF–MS. a Representation of relative intensity of xylo-oligosaccharide after xylanases digestion calculated from raw spectra of b wild type, c fah1-2, d 35S:AnAXE1, and e HrGHypAc, respectively. Figure S3. a ABSL content after pectin extraction in sequential extraction, b ABSL content after xylanase digestion in sequential extraction, c ABSL content after saccharification in sequential extraction. d Xylose extractability through glycome antibody profiling in 1 M KOH, 4 M KOH samples and their combined representation. Data represents mean ± SE, n = 3 biological replicates, Student’s t test at ***p ≤ 0.01, **p ≤ 0.05, * p ≤ 0.1. Figure S4. Proposed model based on xylan digestibility experiment that explains WT xylan accessibility is limited because of hyper-acetylation, and the presence of abundant S lignin. fah1-2 and 35S:AnAXE1 xylan accessibility is increased because of less proximity between lignin–xylan and deacetylation of xylan, respectively. Enhancement in HrGHypAc xylan accessibility is because of the slight decrease in xylan acetylation and G lignin abundance. HrGHypAc lines deposits more cellulose because of change in cell wall integrity. [file 13068_2024_2513_MOESM1_ESM.pptx]

## Slide 1
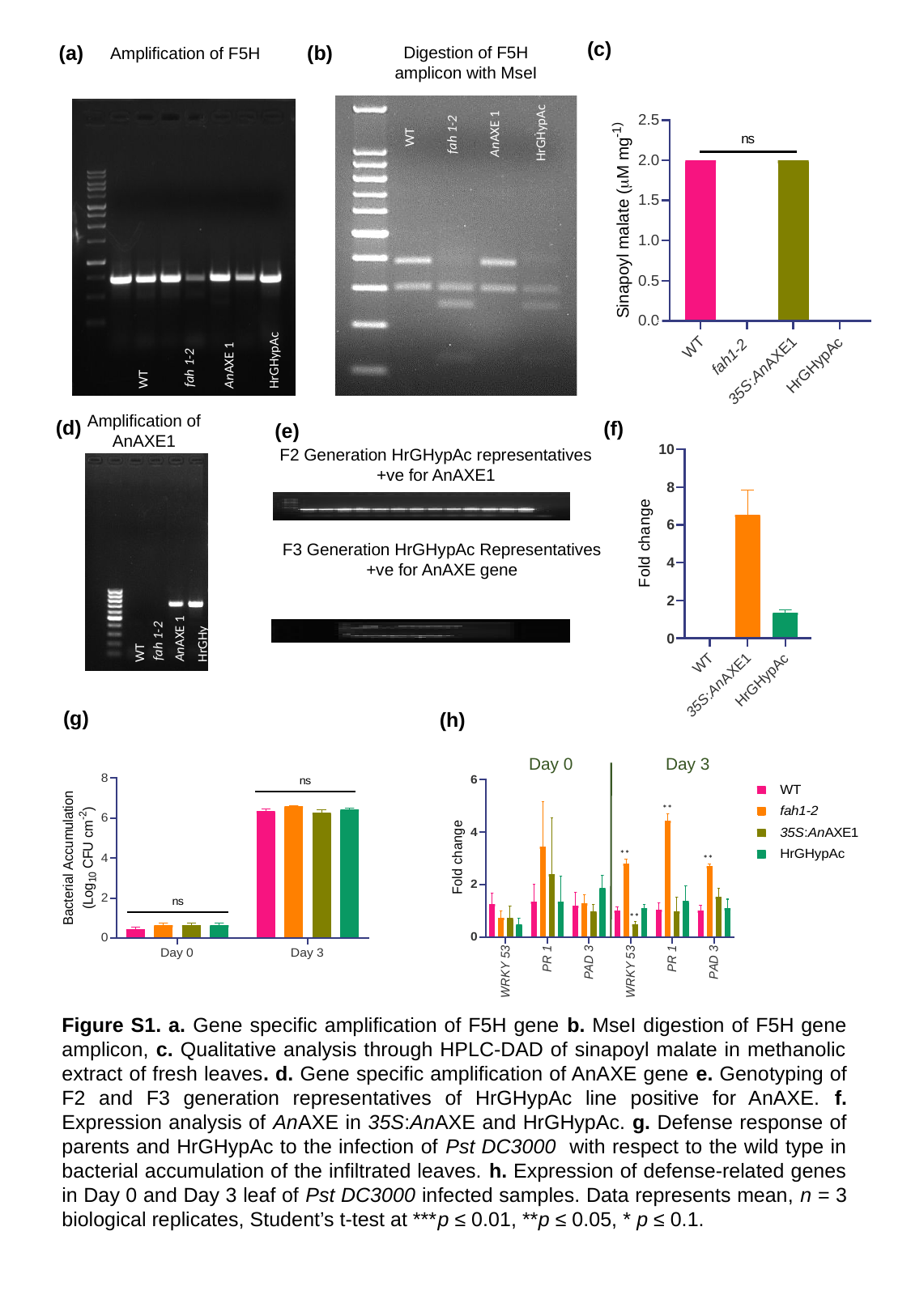

AnAXE 1
HrGHypAc
fah 1-2
WT
(c)
(b)
(a)
Digestion of F5H amplicon with MseI
Amplification of F5H
AnAXE 1
fah 1-2
HrGHypAc
WT
fah 1-2
AnAXE 1
fah 1-2
HrGHypAc
WT
Amplification of
AnAXE1
(d)
(f)
(e)
F2 Generation HrGHypAc representatives +ve for AnAXE1
F3 Generation HrGHypAc Representatives +ve for AnAXE gene
AnAXE 1
fah 1-2
HrGHypAc
WT
(g)
(h)
Day 0
Day 3
**
**
**
**
Figure S1. a. Gene specific amplification of F5H gene b. MseI digestion of F5H gene amplicon, c. Qualitative analysis through HPLC-DAD of sinapoyl malate in methanolic extract of fresh leaves. d. Gene specific amplification of AnAXE gene e. Genotyping of F2 and F3 generation representatives of HrGHypAc line positive for AnAXE. f. Expression analysis of AnAXE in 35S:AnAXE and HrGHypAc. g. Defense response of parents and HrGHypAc to the infection of Pst DC3000 with respect to the wild type in bacterial accumulation of the infiltrated leaves. h. Expression of defense-related genes in Day 0 and Day 3 leaf of Pst DC3000 infected samples. Data represents mean, n = 3 biological replicates, Student’s t-test at ***p ≤ 0.01, **p ≤ 0.05, * p ≤ 0.1.

## Slide 2
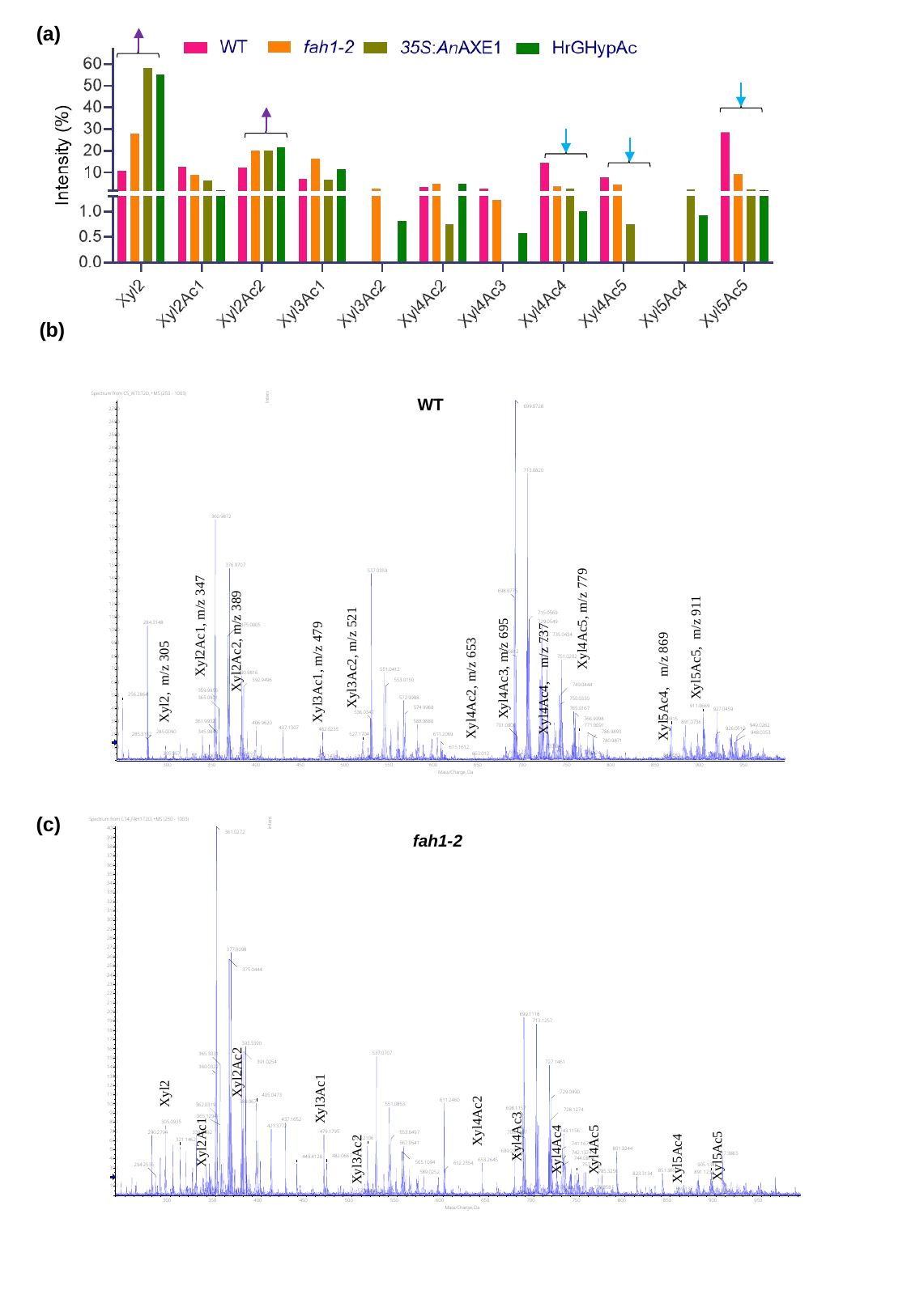

(a)
(b)
WT
Xyl4Ac5, m/z 779
Xyl2Ac1, m/z 347
Xyl2Ac2, m/z 389
Xyl3Ac2, m/z 521
Xyl5Ac5, m/z 911
Xyl3Ac1, m/z 479
Xyl4Ac3, m/z 695
Xyl4Ac4, m/z 737
Xyl2, m/z 305
Xyl4Ac2, m/z 653
Xyl5Ac4, m/z 869
(c)
fah1-2
Xyl2Ac2
Xyl2
Xyl3Ac1
Xyl4Ac2
Xyl4Ac3
Xyl2Ac1
Xyl4Ac4
Xyl4Ac5
Xyl5Ac5
Xyl5Ac4
Xyl3Ac2

## Slide 3
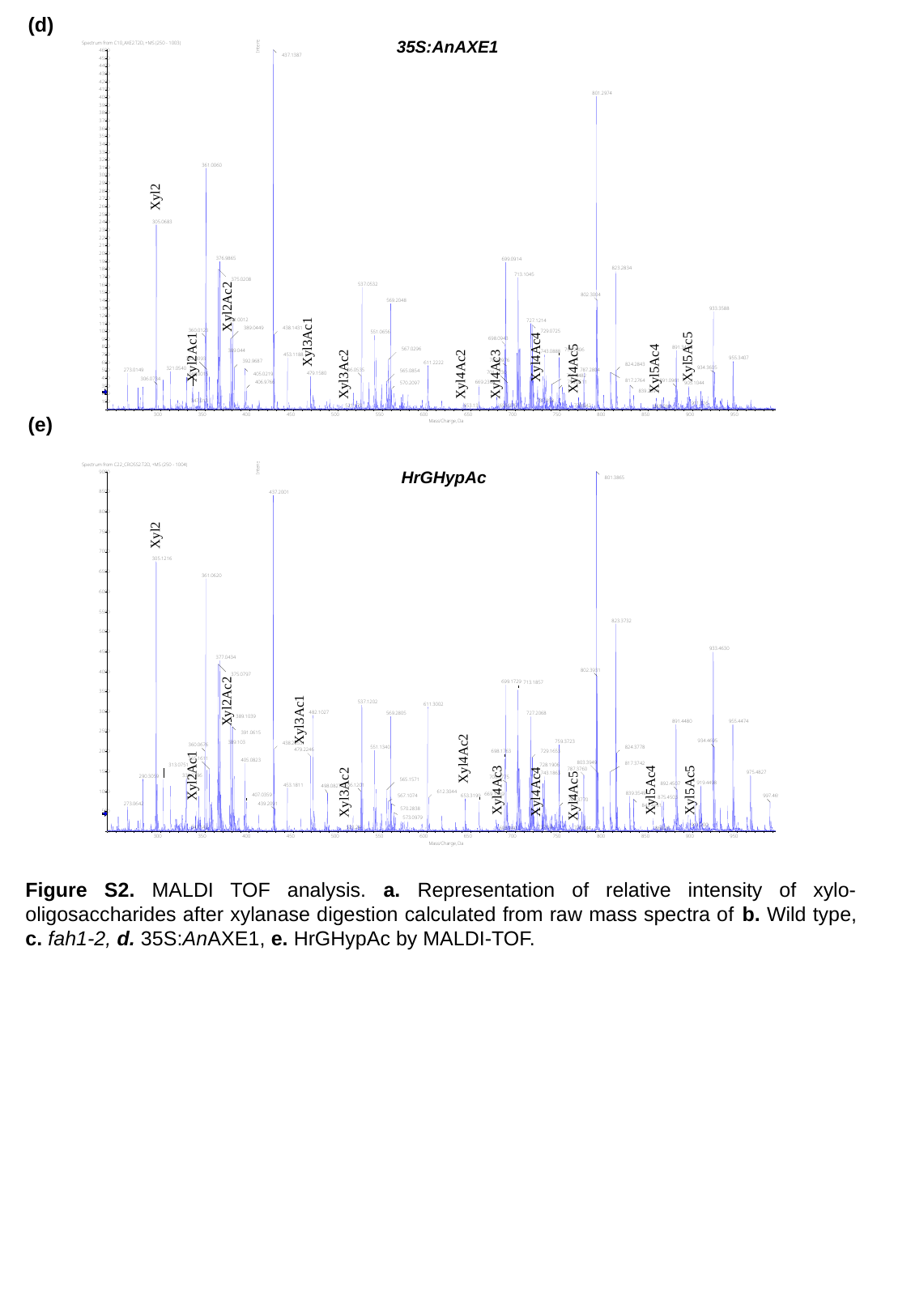

(d)
35S:AnAXE1
Xyl2
Xyl2Ac2
Xyl3Ac1
Xyl2Ac1
Xyl4Ac4
Xyl5Ac5
Xyl4Ac5
Xyl5Ac4
Xyl4Ac3
Xyl4Ac2
Xyl3Ac2
(e)
HrGHypAc
Xyl2
Xyl2Ac2
Xyl3Ac1
Xyl4Ac2
Xyl2Ac1
Xyl5Ac4
Xyl5Ac5
Xyl4Ac3
Xyl4Ac4
Xyl3Ac2
Xyl4Ac5
Figure S2. MALDI TOF analysis. a. Representation of relative intensity of xylo-oligosaccharides after xylanase digestion calculated from raw mass spectra of b. Wild type, c. fah1-2, d. 35S:AnAXE1, e. HrGHypAc by MALDI-TOF.

## Slide 4
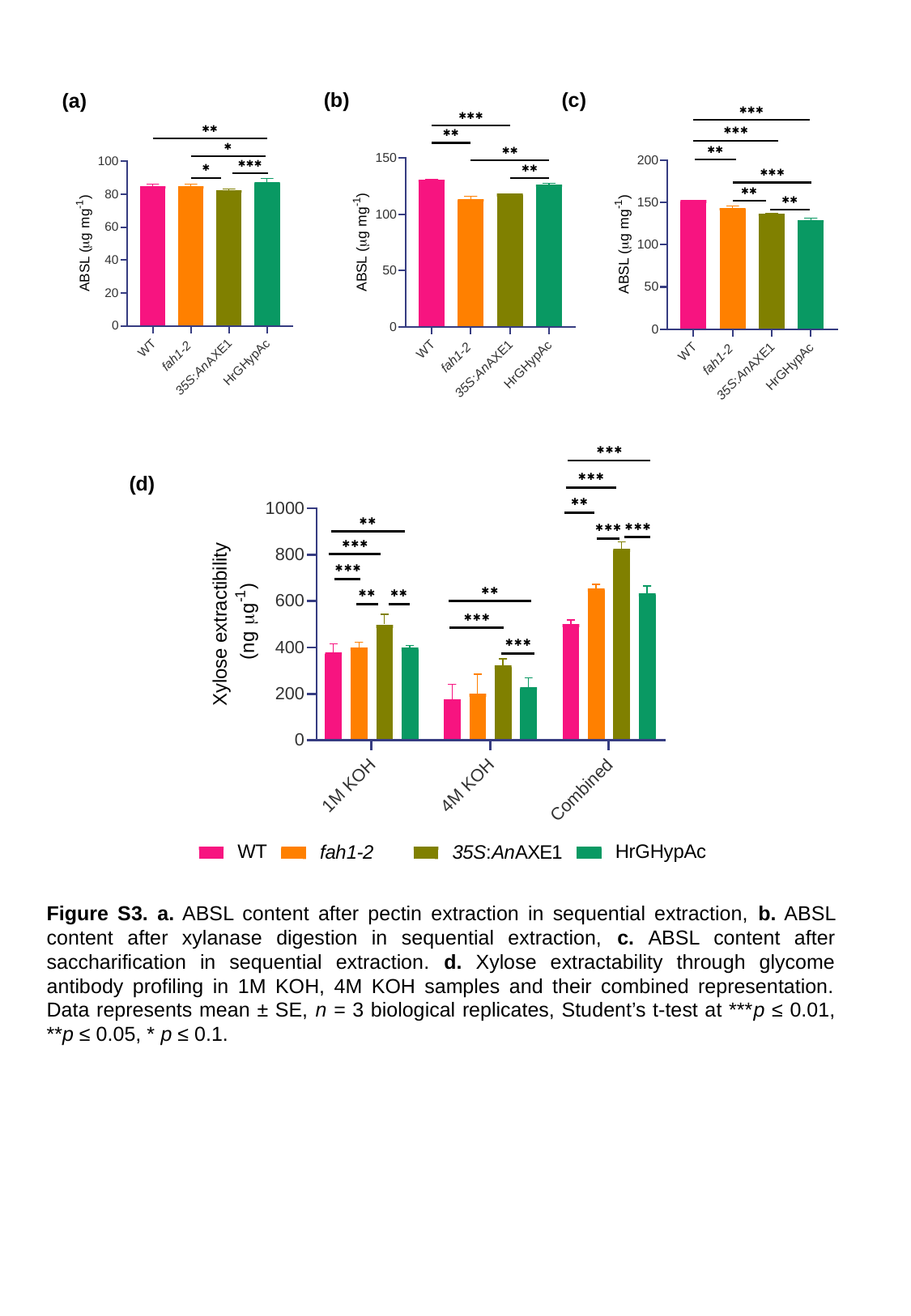

(b)
(c)
(a)
(d)
Figure S3. a. ABSL content after pectin extraction in sequential extraction, b. ABSL content after xylanase digestion in sequential extraction, c. ABSL content after saccharification in sequential extraction. d. Xylose extractability through glycome antibody profiling in 1M KOH, 4M KOH samples and their combined representation. Data represents mean ± SE, n = 3 biological replicates, Student’s t-test at ***p ≤ 0.01, **p ≤ 0.05, * p ≤ 0.1.

## Slide 5
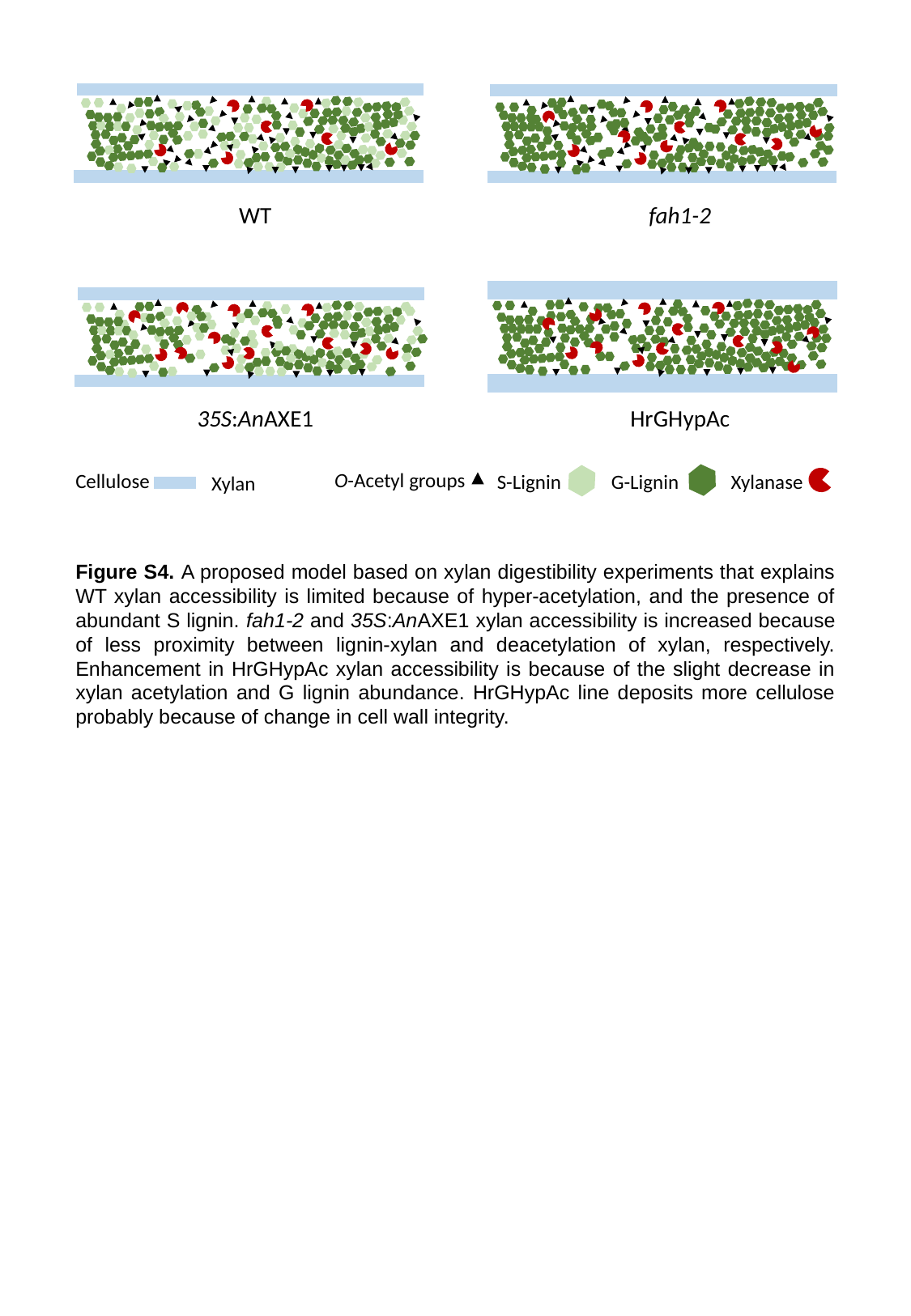

WT
fah1-2
HrGHypAc
35S:AnAXE1
O-Acetyl groups
Cellulose
Xylanase
S-Lignin
G-Lignin
Xylan
Figure S4. A proposed model based on xylan digestibility experiments that explains WT xylan accessibility is limited because of hyper-acetylation, and the presence of abundant S lignin. fah1-2 and 35S:AnAXE1 xylan accessibility is increased because of less proximity between lignin-xylan and deacetylation of xylan, respectively. Enhancement in HrGHypAc xylan accessibility is because of the slight decrease in xylan acetylation and G lignin abundance. HrGHypAc line deposits more cellulose probably because of change in cell wall integrity.
