# Supplementary material for: Modifying lignin composition and xylan O-acetylation induces changes in cell wall composition, extractability, and digestibility
Source: Biotechnol Biofuels Bioprod. 2024 May 31;17:73. doi: 10.1186/s13068-024-02513-5 (PMC11141020; doi:10.1186/s13068-024-02513-5)
Supplement: Supplementary file 2 — Additional file 2: Table S1. Primers used for genotyping of fah1-2 and AnAXE1 and gene expression analysis. [file 13068_2024_2513_MOESM2_ESM.pptx]

## Slide 1
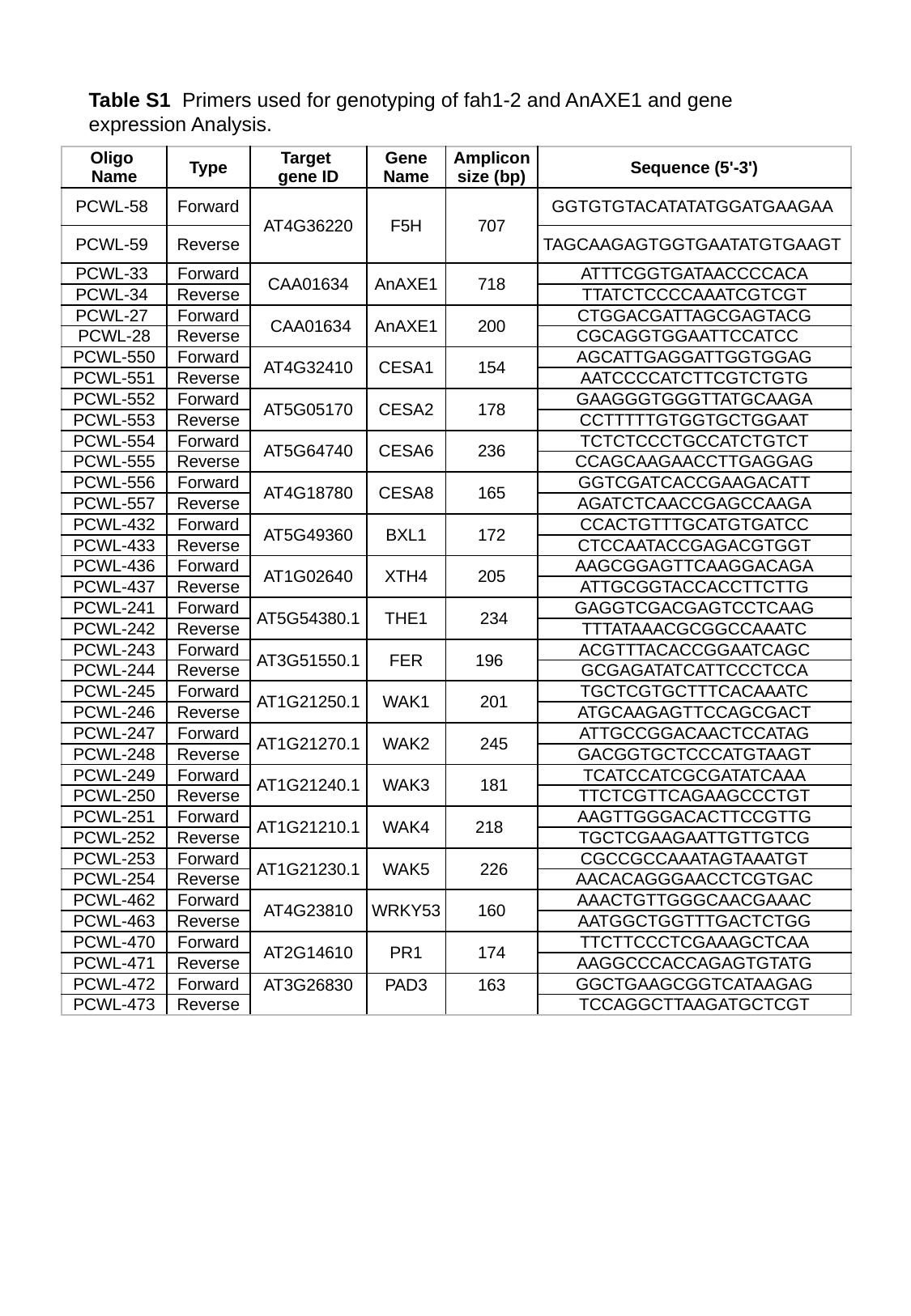

Table S1 Primers used for genotyping of fah1-2 and AnAXE1 and gene expression Analysis.
| Oligo Name | Type | Target gene ID | Gene Name | Amplicon size (bp) | Sequence (5'-3') |
| --- | --- | --- | --- | --- | --- |
| PCWL-58 | Forward | AT4G36220 | F5H | 707 | GGTGTGTACATATATGGATGAAGAA |
| PCWL-59 | Reverse | | | | TAGCAAGAGTGGTGAATATGTGAAGT |
| PCWL-33 | Forward | CAA01634 | AnAXE1 | 718 | ATTTCGGTGATAACCCCACA |
| PCWL-34 | Reverse | | | | TTATCTCCCCAAATCGTCGT |
| PCWL-27 | Forward | CAA01634 | AnAXE1 | 200 | CTGGACGATTAGCGAGTACG |
| PCWL-28 | Reverse | | | | CGCAGGTGGAATTCCATCC |
| PCWL-550 | Forward | AT4G32410 | CESA1 | 154 | AGCATTGAGGATTGGTGGAG |
| PCWL-551 | Reverse | | | | AATCCCCATCTTCGTCTGTG |
| PCWL-552 | Forward | AT5G05170 | CESA2 | 178 | GAAGGGTGGGTTATGCAAGA |
| PCWL-553 | Reverse | | | | CCTTTTTGTGGTGCTGGAAT |
| PCWL-554 | Forward | AT5G64740 | CESA6 | 236 | TCTCTCCCTGCCATCTGTCT |
| PCWL-555 | Reverse | | | | CCAGCAAGAACCTTGAGGAG |
| PCWL-556 | Forward | AT4G18780 | CESA8 | 165 | GGTCGATCACCGAAGACATT |
| PCWL-557 | Reverse | | | | AGATCTCAACCGAGCCAAGA |
| PCWL-432 | Forward | AT5G49360 | BXL1 | 172 | CCACTGTTTGCATGTGATCC |
| PCWL-433 | Reverse | | | | CTCCAATACCGAGACGTGGT |
| PCWL-436 | Forward | AT1G02640 | XTH4 | 205 | AAGCGGAGTTCAAGGACAGA |
| PCWL-437 | Reverse | | | | ATTGCGGTACCACCTTCTTG |
| PCWL-241 | Forward | AT5G54380.1 | THE1 | 234 | GAGGTCGACGAGTCCTCAAG |
| PCWL-242 | Reverse | | | | TTTATAAACGCGGCCAAATC |
| PCWL-243 | Forward | AT3G51550.1 | FER | 196 | ACGTTTACACCGGAATCAGC |
| PCWL-244 | Reverse | | | | GCGAGATATCATTCCCTCCA |
| PCWL-245 | Forward | AT1G21250.1 | WAK1 | 201 | TGCTCGTGCTTTCACAAATC |
| PCWL-246 | Reverse | | | | ATGCAAGAGTTCCAGCGACT |
| PCWL-247 | Forward | AT1G21270.1 | WAK2 | 245 | ATTGCCGGACAACTCCATAG |
| PCWL-248 | Reverse | | | | GACGGTGCTCCCATGTAAGT |
| PCWL-249 | Forward | AT1G21240.1 | WAK3 | 181 | TCATCCATCGCGATATCAAA |
| PCWL-250 | Reverse | | | | TTCTCGTTCAGAAGCCCTGT |
| PCWL-251 | Forward | AT1G21210.1 | WAK4 | 218 | AAGTTGGGACACTTCCGTTG |
| PCWL-252 | Reverse | | | | TGCTCGAAGAATTGTTGTCG |
| PCWL-253 | Forward | AT1G21230.1 | WAK5 | 226 | CGCCGCCAAATAGTAAATGT |
| PCWL-254 | Reverse | | | | AACACAGGGAACCTCGTGAC |
| PCWL-462 | Forward | AT4G23810 | WRKY53 | 160 | AAACTGTTGGGCAACGAAAC |
| PCWL-463 | Reverse | | | | AATGGCTGGTTTGACTCTGG |
| PCWL-470 | Forward | AT2G14610 | PR1 | 174 | TTCTTCCCTCGAAAGCTCAA |
| PCWL-471 | Reverse | | | | AAGGCCCACCAGAGTGTATG |
| PCWL-472 | Forward | AT3G26830 | PAD3 | 163 | GGCTGAAGCGGTCATAAGAG |
| PCWL-473 | Reverse | | | | TCCAGGCTTAAGATGCTCGT |
